# Health-Related Quality of Life in Relation to Fruit and Vegetable Intake among Polish Pharmacists

**DOI:** 10.3390/healthcare10050930

**Published:** 2022-05-18

**Authors:** Magdalena Kurnik-Łucka, Dominika Grońska, Marcin Wojnarski, Paweł Pasieka, Elżbieta Rząsa-Duran, Krzysztof Gil

**Affiliations:** 1 Department of Pathophysiology, Jagiellonian University Medical College, 31-008 Kraków, Poland; dominika.gronska@student.uj.edu.pl (D.G.); marcin-wojnarski@wp.pl (M.W.); ppasieka96@outlook.com (P.P.); krzysztof.m.gil@uj.edu.pl (K.G.); 2 Pharmacists’ Chamber of Lesser Poland, 31-238 Kraków, Poland; duranela@poczta.onet.pl

**Keywords:** workplace nutrition, HQoL, KomPAN questionnaire, RAND-SF-36 questionnaire

## Abstract

A direct association between health-related quality of life (HQoL) and nutrition remains unclear, although lifestyle habits are known to impact on human health. Thus, the aim of this study was to estimate an association between dietary habits, such as vegetable and fruit consumption, in particular, and HQoL. SF-36 (RAND-SF-36) and the Dietary Habits and Nutrition Beliefs Questionnaire (KomPAN) were addressed to Polish pharmacists with a valid professional license. After the preliminary verification, 667 pharmacists were included into the study, and altogether, 11 questions from the KomPAN questionnaire and all RAND-SF-36 multiple choice questions were processed for statistical analysis. All HQoL scores, excluding physical functioning and role-physical, were significantly higher in the group declaring fruit consumption ≥1 time per day (*p* < 0.005), while physical functioning (*p* = 0.008) and general health (*p* = 0.03) were significantly higher in the group declaring vegetable consumption ≥1 time per day. Thus, there is indeed a positive association between vegetable and fruit consumption and HQoL. Increased fruit intake could certainly impact on the health-related quality of life of Polish pharmacists, primarily in terms of mental functioning, while increased vegetable intake could impact in terms of physical functioning.

## 1. Introduction

The latest report (2019) of the Supreme Audit Office (SAO) on the healthcare system in Poland shows that there is an urgent need for organisational changes. The importance of investing in the health of citizens through education and health prevention has been emphasised [1]. According to the Lalonde’s concept (also known as The Health Field Concept), lifestyle has the greatest impact on human health [2], and diet remains a key component of lifestyle. In Poland, following the guidelines of the National Centre for Nutrition Education, the food group that most significantly lowers the risk of lifestyle-associated diseases is vegetables, followed by fruit [3]. The recent meta-analysis (2017) associated high intake of fruit and vegetables with a reduced risk of cardiovascular disease and cancers, among other diseases [4], which is consistent with similar previous studies [5,6]. Authors estimated that 5.6 and 7.8 million premature deaths worldwide in 2013 could have resulted from fruit and vegetable intake below the recommended ranges [4]. Moreover, recent results from two prospective cohort studies of US men and women and a meta-analysis of 26 cohort studies associated higher intake of fruit and vegetable with lower mortality, and the risk reduction plateaued at around five servings of fruit and vegetables per day [7].

It is widely recognised that a person’s quality of life is closely correlated with one’s health status, and this so-called health-related quality of life (HQoL) should be further associated with nutrition. However, our understanding of this association remains incomplete, but both over- and malnutrition are known to be significantly associated with numerous disorders [8]. The potential role of pharmacists in patients’ nutrition education has been numerously emphasised [9,10,11,12]. Adequate nutrition knowledge does not guarantee positive changes in eating behaviour [13]; however, self-awareness of the importance of healthy dietary habits might be the first step towards eating behaviour improvement [14]. On the other hand, Kelly et al. stressed that medical personnel with poor dietary habits were characterised by poorer physical and psychological well-being [15], while Almogbel emphasised that pharmacists’ occupational stress has a negative impact on their quality of life, which consequently could have a potential role in perpetuating poor dietary habits [16]. Hence, there is a perceived and continuous need to also implement occupational health promotion programs for healthcare workers [17,18].

Data regarding a direct association between the frequency of fruit and vegetable intake and HQoL in the healthcare workforce, including Polish and non-Polish pharmacists, is limited. However, based on indications from the general population, it is likely that better nutrition habits should be associated with a better HQoL [19,20,21,22,23]. Thus, the aim of the present study was to estimate, using validated questionnaire tools, an association between dietary habits, such as vegetable and fruit consumption, in particular, and health-related quality of life, with Polish pharmacists as the study population.

## 2. Materials and Methods

### 2.1. Study Sample, Recruitment and Data Collection

The study sample was recruited between May and October 2018. A Polish translation of SF-36 [24,25], consistent with the Medical Outcomes Study 36-Item Short Form Health Survey (RAND-SF-36), and the Dietary Habits and Nutrition Beliefs Questionnaire (KomPAN) [26,27] were made available at Jagiellonian University Medical College domain “http://ankiety.cm-uj.krakow.pl (accessed on 28 October 2018)” and were addressed to Polish pharmacists with a valid professional license in Poland. Participation in the study was voluntary and anonymous. Information about the study was published on the website of the Pharmacists’ Chamber of Lesser Poland and the portal E-farmacja.pl. The study protocol was approved by the Jagiellonian University Bioethics Committee (consent number–1072/6120/197/2017). The study was a post hoc analysis of two previous studies aiming to separately assess nutrition knowledge and habits [12], and the health-related quality of life among Polish pharmacists [28].

### 2.2. The SF-36 (RAND-SF-36) Health Survey

The Medical Outcomes Study 36-Item Short Form Health Survey (RAND-SF-36) questionnaire is a validated survey instrument used to assess health-related quality of life (HQoL) scores, with the two major health domains—physical summary score (PSS) and mental summary score (MSS). The PSS is represented by four subdomains: (1) physical functioning, which describes limitations of basic physical activities (e.g., walking) due to health impairment; (2) role-physical, which is concerned with difficulties in performing work or daily activities due to poor physical health; (3) bodily pain, which describes presence of pain and limitations imposed by it; (4) general health, based on subjective evaluation of respondent’s health. The MSS is represented by four subdomains: (1) vitality, which describes the presence of energy and fatigue; (2) social functioning, which describes limitations in social activities due to health problems; (3) role-emotional, which describes difficulties with performing work or daily activities due to emotional problems; (4) mental health, which describes the presence of agitation and/or depression. The raw score of each of the eight SF-36 (RAND-SF-36) dimensions is derived by summing the item scores and converting them to the 0–100 point scale, and a low numerical value indicates a negative perception, while a high numerical value indicates a positive perception of one’s health [24,25]. Finally, the data from SF-36 questionnaire were collated with the selected questions from the KomPAN questionnaire.

### 2.3. The KomPAN Questionnaire

The Dietary Habits and Nutrition Beliefs Questionnaire (KomPAN) for people aged 15–65, designed by the Behavioural Nutrition Team, Committee of Human Nutrition, Polish Academy of Sciences, is a validated tool designed to assess nutrition habits in the Polish population [26,27,29]. The self-administered version of the questionnaire was used to collect data related to food frequency consumption together with basic demographic and personal data (including self-reported anthropometric measurements). To assess the relationship between health-related quality of life (HQoL) and fruit and vegetable consumption, answers to the following questions were taken into consideration: (1) “How often do you eat fruit?”; (2) How often do you eat vegetables?”.

### 2.4. Sample Size and Statistical Analysis

Sample size was calculated based on the Chebyshev approximation formula. With 99% level of confidence, 5% margin of error, and since there are 34,573 nationally registered pharmacists in May 2018 in Poland, at least 653 respondents were needed for the study. The total study sample consisted of 1412 respondents. In the initial analysis performed with Microsoft Excel for Mac 2011 software (version 14.7.2 Microsoft Corporation, Jagiellonian University license, Redmond, WA, USA) respondents were excluded for the following reasons: (1) incomplete data (*n* = 647); (2) failure to meet the age criterion, i.e., age over 65 years—for the KomPAN questionnaire (*n* = 13); (3) failed systemic verification of respondents’ reliability based on the KomPAN manual quide (*n* = 85). Consequently, 667 pharmacists were included into the study.

Statistical analysis was performed with Statistica 13.3 software (TIBCO Software Inc., Palo Alto, CA, USA; Jagiellonian University license). Data were presented as percentages of the sample for categorical variables and median together with means and standard deviations (SD) for continuous variables. Distributions were evaluated for normality with the Shapiro–Wilk test. The distribution of all analysed scores did not meet the criteria of normality and, thus, non-parametric tests (Mann–Whitney U test) were applied. The Spearman’s rank correlation coefficients were calculated between HQoL scores and fruit and vegetable consumption. The results of all tests were only considered statistically significant when *p*-values were below 0.05. The presentation of results was carried out with adherence to the STROBE statements [30].

## 3. Results

### 3.1. Baseline Data

The study included 667 pharmacists. There were 573 (85.9%) women and 94 (16.1%) men in the sample. Normal weight (according to body mass index calculated based on voluntary information regarding weight and height, *n* = 650) was found in 62.6% of pharmacists, and within those respondents almost three-quarters had a waist circumference within normal range (below 80 cm and 94 cm in females and males, respectively). Moderate and high recreational physical activity was reported by 68.2% respondents (48.6% and 19.6%, respectively). The majority of respondents were current non-smokers (94.0%) and the majority reported no history of smoking (74.2%). The minority of respondents reported no alcohol consumption (18.6%).

### 3.2. Dietary Patterns

Over two-fifths of respondents (43.5%) declared that they consume 4 meals per day, yet only 23.9% reported eating all of their meals in a set daily schedule (Table 1). Only 4.1% of respondents never ate snacks (3.7% of females and 6.5% of males), while almost two-fifths (40.6% of females and 30.1% of males) declared that they snack ≥1 time per day. Fruit was the preferred snack in the sample. The majority of pharmacists consumed both fruit and vegetables ≥ 1 time per day (Table 2 and Table 3).

To assess the frequency of consumption, respondents could choose one of six categories: never, 1–3 times a month, once a week, few times a week, once a day, or a few times a day. However, because some of the answers were chosen infrequently, responses were grouped according to the frequency of consumption of either less than once or equal and/or more than once per day. The answers to these questions are shown in Table 2.

The highest proportion of those who declared that they eat vegetables ≥1 time per day was observed among men in the age range of 60–65 years of age, while the highest proportion of those who declared that they eat fruit ≥1 time per day was observed among woman in the age range of 51–60 years of age. However, the minority of respondents eat canned or pickled vegetables as well as foods made from pulses, such as beans, peas, soybeans, lentils ≥1 time per day (Table 3).

### 3.3. Fruit and Vegetables Intake and Health-Related Quality of Life

The analysis revealed a difference in health-related quality of life in both physical and mental health domains, in the relation to fruit consumption. HQoL scores, excluding physical functioning and role-physical, were significantly higher in the group declaring fruit consumption ≥1 time per day (Table 4). Conversely, mental component scores (vitality, role-emotional, social functioning, and mental health) were indifferent in relation to more frequent vegetable consumption (Table 5). HQoL scores such as physical functioning and general health were significantly higher in the group declaring vegetable consumption ≥ 1 time per day. The Spearman’s rank correlation analysis showed correlations, although they were weak, between fruit consumption and mental summary score (r_s_ = 0.17, *p* < 0.001) as well as vegetable consumption and physical summary score (r_s_ = 0.13, *p* < 0.001). What is more, respondents who chose vegetables as the preferred snack had significantly higher mean mental (77.6 ± 15.2, *p* = 0.001) and physical (73.6 ± 13.7, *p* = 0.02) summary scores in comparison to the rest of the study population (71.8 ± 17.9 and 71.9 ± 12.9, respectively).

## 4. Discussion

The multidimensional concept of health-related quality of life comprises those aspects of overall quality of life that can affect either physical or mental health [31]. Numerous instruments have been developed to measure this, including the Medical Outcomes Study 36-Item Short Form Health Survey (SF-36) to evaluate the health-related quality of life [24,25,32]. Our results showed a significant association between more frequent consumption of fruit and better physical and mental HQoL among Polish pharmacists. So far, many studies have indicated that consumption of plant-derived foods should indeed improve the quality of life in all its indicators [19,20]. Lately, Conner et al. also reported that consumption of fresh fruit and vegetables among young adults should provide psychological benefits in particular, even in the short term [21]. Similarly, diets rich in fruit and vegetables were associated with lower psychological stress in young Saudi women [22]. However, our results modestly related more frequent consumption of vegetables with higher scores in physical functioning and general health subdomains only.

However, our respondents declared generally higher consumption of both fruit and vegetables in comparison to the Polish population average and relatively high in comparison to populations of several European countries (Table 6).

It may be assumed that Polish pharmacists’ nutritional habits at least partially correspond with current trends of the plant-based diet [34]. The diet was proposed in 2019 by researchers of the EAT-Lancet Commission on Food, Planet, and Health. This nutrition model indicates that the daily ratio should include a variety of plant products, which in practice means mainly the consumption of fruit and vegetables, pulses, cereal products, nuts, and seeds [34,35]. It is important to note that this model of nutrition is highly recommended not only for health reasons but also for environmental (for example, greenhouse gas emissions coming from ovolactovegetarian and vegan diets are estimated to be around 35% and around 50% lower, respectively, than omnivore diets [36]) and economic reasons [37]. Good nutrition knowledge, which we previously reported among Polish pharmacists [12], together with satisfactory eating behaviours, should also lay the foundations for successful nutrition education of patients.

There are numerous benefits of including fruit and vegetables in the diet. Most of them are potent sources of fibre, vitamins (especially C and A), electrolytes (especially potassium), and antioxidants, while being generally low in energy density. Fruit juices and potatoes are usually contained in separate nutritional categories, because of dietary directives to eat whole fruits and minimise consumption of foods high in fat and sodium (e.g., French fries). Although a boiled potato is a nutrient-dense food, a fried potato may constitute a substantial amount of fat and sodium to a diet [23]. In our study, only 5.4% of respondents declared eating potatoes ≥ 1 time per day (excluding French fries and chips). In Poland, considering the amount of consumption, the sources of dietary fibre are mainly cereal products, which contribute about 54% of this component, whereas vegetables and potatoes together contribute about 33% [38]. On the other hand, processing may even increase the fibre content of a product by removal of water. Fruit juices are not entirely devoid of fibre, and for example in the United Kingdom, 1 glass (150 mL) of fruit juice counts as one portion, but juice can only count as a maximum of 1 portion per day [23]. According to WHO/FAO recommendations, an intake of 25 g of fibre per day allows the body to function properly [39]. However, recent studies show that the average dietary fibre intake in Poland is 17.5 g/person/day in women and 20.9 g/person/day in men [40]. Dietary fibre intake provides health benefits related to intestinal function, maintenance of or reduction in blood cholesterol levels, modulation of the postprandial glycaemic response [41,42,43] or protection against various diseases [44,45], including cancers [46,47]. In our study, over 73% and 66% of pharmacists declared vegetable and fruit intake ≥1 time per day, respectively; however, the majority of respondents declared <1 per day consumption of canned or pickled vegetables as well as foods made from pulses, such as beans, peas, soybeans, lentils. Thus, overall eating habits may partly explain weak association between vegetable intake and general health as well as physical functioning domains only (with no association with other subdomains) despite the frequent consumption of vegetables in our study population.

Among our respondents, 40.6% of female and 30.1% of male pharmacists declared that they snack ≥1 time per day, and for almost half of those who snack, this included the consumption of sweet snacks, e.g., candies, cookies, cakes, chocolate bars, muesli bars, waffles. Unhealthy, energy-dense sugary and salty snacks are known to negatively impact on individuals’ well-being [48]. Chiou et al. reported that participation in health promotion activities was related to healthier behaviours among hospital staff, but the level of participation was generally low among nurses, pharmacists, and physicians [17]. According to scientific evidence, work-related outcomes can be positively influenced through more nuanced health promotion efforts (including workplace nutrition) [49].

It is also worth noticing that pharmacists are vulnerable to professional burnout, and their professional quality of life has been found to be relatively low, especially in comparison to other medical professionals [12,16,50]. The governmental workplace programs for pharmacists might positively contribute to their quality of work-life [51]. Previously, we reported a relatively low mental HQoL among pharmacists, with the lowest median scores of the mental health domain among the youngest (below 30) respondents and 51- to 60-year-olds [28]. Thus, considering a positive association between frequent intake of fruit and vegetables and all subdomains of the mental summary score, pharmacists should benefit from a regular and additional intake of fresh, high-quality fruit and vegetables at their workplace.

### Limitations and Strengths of the Study

The KomPAN questionnaire is a qualitative food frequency questionnaire only, which limits its interpretation. What is more, the self-reported version used in our study is prone to inaccuracy, and reported values are subject to systematic (intake-related and person-specific) as well as random errors [52]. Our analysis is only an illustration of some of the trends related to the eating habits of Polish pharmacists. The length of the entire survey with two self-reported versions of questionnaires resulted in diminished sample size due to incomplete data, and likely reduced the accuracy of the assessment. Detailed subtypes of fruits and vegetables and their serving sizes were not determined in our study, but attempts to link any particular food or nutrient to health or disease are generally limited. Thus, to assess direct health benefits or the health-related quality of life in relation to fruit and vegetable intake, prospective cohort studies would be most preferable. However, to our knowledge, this is the first study to examine associations between fruit and vegetable intake in relation to health-related quality of life among Polish pharmacists, using validated tools such as a culture-specific nutrition questionnaire [26,27] and RAND-SF-36 [24,25].

## 5. Conclusions

Our results show that frequent fruit intake (i.e., ≥1 time per day) has a potential to improve the health-related quality of life of Polish pharmacists, both in terms of their mental and—to a lesser extent—physical summary scores. Meanwhile, increased vegetable intake (i.e., ≥1 time per day) should exclusively alter the physical summary score. Thus, pharmacists should benefit from a regular and additional intake of fresh, high-quality fruit and vegetables. Access (or a free access as one of employees’ benefits) to fresh fruit and vegetables for all healthcare professionals at their workplace should be considered by policy makers to support their health and well-being.

## Figures and Tables

**Table 1:** Dietary patterns among Polish pharmacists (*n* = 667).

Questions from the KomPAN Questionnaire	F (%)	M (%)	*p*-Value	In Total (%)
How many meals do you consume daily?				
1–2	4.3	12.9	0.006	**5.5**
3	25.0	28.0		**25.4**
4	44.9	35.5		**43.5**
5	25.8	23.7		**25.5**
**Do you always eat meal at the same time of the day?**				
no	17.5	15.1	0.2	**17.1**
yes, but only some of them	59.9	53.8		**59.0**
yes, all of them	22.7	31.2		**23.9**
**How often do you snack between meals?**				
never	3.7	6.5	0.45	**4.1**
1–3 times per month	11.9	9.7		**11.6**
once a week	13.6	16.1		**14.0**
few times a week	31.1	37.6		**32.0**
once a day	20.8	14.0		**19.8**
few times a day	19.0	16.1		**18.6**
**What type of food do you usually eat between meals on weekdays?** (a question for those who snack; multiple choice possible)				
fruit	67.0	62.4	0.4	**66.4**
vegetables	17.1	18.3	0.8	**17.3**
unsweetened dairy drinks and desserts, e.g., yogurt, cottage cheese, milk	24.6	22.6	0.7	**24.3**
sweetened dairy drinks and desserts, e.g., yogurt, cottage cheese, milk	11.5	9.7	0.6	**11.3**
sweet snacks, e.g., candies, cookies, cakes, chocolate bars, muesli bars, waffles	49.4	49.5	0.9	**49.4**
salty snacks, e.g., crackers, sticks, chips, fries	17.3	23.7	0.1	**18.2**
nuts, almonds, seeds, pips	55.3	52.7	0.6	**55.0**
other	4.2	3.2	0.7	**4.1**

Abbreviations: F, females, M, males.

**Table 2:** The number of answers in the KomPAN questionnaire with regard to the frequency of consumption of fruit and vegetables (*n* = 667).

Frequency of Consumption	How Often Do You Eat Fruit?		How Often do You Eat Vegetables?		Frequency of Consumption
	Number of Answers				
Never	1	227	0	178	<1 time per day
1–3 times per month	19		7		
Once a week	25		14		
Few times a week	182		157		
Once a day	242	440	201	489	≥1 time per day
Few times a day	198		288		

**Table 3:** Fruit and vegetable intake among Polish pharmacists (*n* = 667) Abbreviations: F, females.

Percentage of the Sample (%)											
Questions from the KomPAN Questionnaire	In Total	Age: ≤30		31–40		41–50		51–60		61–65	
		F	M	F	M	F	M	F	M	F	M
How often do you eat fruit?											
<1 time per day	**34**	39.6	38.9	52.6	40.6	31.0	57.1	23.6	53.3	28.6	25.0
≥1 time per day	**66**	60.4	61.1	47.4	59.4	69.0	42.9	76.4	46.7	71.4	75.0
**How often do you drink fruit juice?**											
<1 time per day	**88.6**	86.1	77.8	89.5	84.4	90.7	85.7	88.7	86.7	92.9	100.0
≥1 time per day	**11.4**	13.9	22.2	10.5	5.6	9.3	14.3	11.3	13.3	7.1	0.0
**How often do you eat vegetables?**											
<1 time per day	**26.7**	27.7	44.4	16.7	31.3	31.0	57.1	25.5	46.7	35.7	12.5
≥1 time per day	**73.3**	72.3	55.6	83.3	68.7	69.0	42.9	74.5	53.3	64.3	87.5
**How often do you eat potatoes (excluding French fries and chips)?**											
<1 time per day	**94.6**	95.1	100.0	97.1	93.8	92.2	95.2	92.5	86.7	92.9	100.0
≥1 time per day	**5.4**	4.9	0.0	2.9	6.2	7.8	4.8	7.5	13.3	7.1	0.0
**How often do you eat canned or pickled vegetables?**											
<1 time per day	**96.6**	95.1	100.0	96.2	96.9	96.9	95.2	97.2	93.3	100.0	100.0
≥1 time per day	**3.4**	4.9	0.0	3.8	3.1	3.1	4.8	2.8	6.7	0.0	0.0
**How often do you drink vegetable or fruit and vegetable juices?**											
<1 time per day	**96.4**	97.0	100.0	99.0	100.0	94.6	95.2	92.5	93.3	92.9	100.0
≥1 time per day	**3.6**	3.0	0.0	1.0	0.0	5.4	4.8	7.5	6.7	7.1	0.0
**How often do you eat foods made from pulses, such as beans, peas, soybeans, lentils?**											
<1 time per day	**97.8**	96.0	100.0	97.1	96.9	100.0	95.2	98.1	100.0	100.0	100.0
≥1 time per day	**2.2**	4.0	0.0	2.9	3.1	0.0	4.8	1.9	0.0	0.0	0.0

**Table 4:** Comparison of HQoL according to SF-36 with respect to fruit intake.

Analysed Domain	Fruit Intake < 1 per Day		Fruit Intake ≥ 1 per Day		*p*-Value
	Median	Mean ± SD	Median	Mean ± SD	
**Physical summary score**	73.8	70.6 ± 13.2	77.5	72.6 ± 13.5	0.003
Physical functioning	100	93.1 ± 11.5	95	93.1 ± 11.6	0.998
Role-physical	100	84.9 ± 30.4	100	86.6 ± 30.5	0.15
Bodily pain	60	57.8 ± 20.9	70	62.3 ± 21.4	0.009
General health	45	46.7 ± 8.1	50	48.3 ± 8.3	0.005
**Mental summary score**	73.3	67.9 ± 19.8	79.1	75.1 ± 16.0	<0.001
Vitality	55	53.4 ± 20.4	66	61.4 ± 17.4	<0.001
Role-emotional	100	78.0 ± 35.8	100	86.2 ± 28.3	0.005
Social functioning	75	76.3 ± 22.7	87.5	82.0 ± 20.3	0.001
Mental health	64	64.7 ± 16.8	72	70.5 ± 15.5	<0.001

**Table 5:** Comparison of HQoL according to SF-36 with respect to vegetable intake.

Analysed Domain	Vegetable Intake < 1 per Day		Vegetable Intake ≥ 1 per Day		*p*-Value
	Median	Mean ± SD	Median	Mean ± SD	
**Physical component score**	75	70.3 ± 14.2	77,5	72.5 ± 13.1	0.01
Physical functioning	95	91.0 ± 13.6	100	93.9 ± 10.7	0.008
Role-physical	100	85.4 ± 30.7	100	86.3 ± 30.5	0.5
Bodily pain	60	57.9 ± 21.5	70	61.8 ± 21.1	0.07
General health	45	46.8 ± 8.9	50	48.2 ± 8.0	0.03
**Mental component score**	77.9	72.1 ± 18.4	78	72.9 ± 17.4	0.85
Vitality	60	55.7 ± 21.3	60	59.9 ± 17.8	0.6
Role-emotional	100	84.7 ± 30.5	100	83.0 ± 31.6	0.5
Social functioning	87.5	79.9 ± 21.1	87.5	80.2 ± 21.4	0.9
Mental health	68	66.7 ± 17.5	72	69.2 ± 15.6	0.2

**Table 6:** Daily consumption of fruit and vegetables in some of the European Union * countries, 2017 [33].

	Latvia	Hungary	Bulgaria	Romania	Poland	Belgium	Ireland	Portugal	Italy
**Vegetables**	44%	30%	45%	41%	61%	84%	84%	78%	80%
**Fruit**	35%	40%	37%	42%	58%	61%	64%	81%	85%

* In three Member States, less than 40% of the population ate fruit on a daily basis: Latvia, Bulgaria and Lithuania (37%). In most Member States, between 50% and 80% of the population reported to eat vegetables daily, but there were five Member States where the proportion was below 50%: Hungary, Romania, Latvia, Lithuania (45%) and Bulgaria.

## Data Availability

The data are available from the corresponding author upon reasonable request.
